# Associations between Suicidal Ideation and Relatives’ Physical and Mental Health among Community Residents: Differences between Family Members and Lineal Consanguinity

**DOI:** 10.3390/ijerph192315997

**Published:** 2022-11-30

**Authors:** Caifeng Li, Zhen Wei, Yifan Wang, Long Sun

**Affiliations:** 1 Centre for Health Management and Policy Research, School of Public Health, Cheeloo College of Medicine, Shandong University, Jinan 250012, China; 2 National Health Commission of China (NHC) Key Laboratory of Health Economics and Policy Research, Shandong University, Jinan 250012, China

**Keywords:** suicidal ideation, family, risk factors, logistic models

## Abstract

(1) Background: Despite the verified relationship between relatives’ characteristics and individual suicidal ideation, few studies have discussed the role of family members and lineal consanguinity independently according to whether they live together with the individuals or not. (2) Methods: The data in this study were collected in November 2019 and identified rural adults over 18 years old in Shandong as the survey objects, with a total of 879 valid cases included in this survey. Logistic regression analysis was employed to examine the risk factors affecting adults’ suicidal ideation and differentiate the effects of a family member and lineal consanguinity’s physical and mental health. Relatives’ physical and mental health were estimated by three aspects: whether they were suffering from chronic diseases, mental illness, or alcoholism. (3) Results: The study showed that a family member’s physical (OR = 2.303, *p* < 0.01) and mental health (OR = 5.877, *p* < 0.05) was related to suicidal ideation, but the association between lineal consanguinities’ physical and mental health and suicidal ideation were not supported. People over 40 years old (OR = 6.528, *p* < 0.05), from only-child families (OR = 4.335, *p* < 0.01), with household indebtedness (OR = 2.992, *p* < 0.001), or difficulty falling asleep (OR = 3.165, *p* < 0.001) had risk factors of suicidal ideation. (4) Conclusions: The physical and mental health of individuals’ family members are related to their suicidal ideation, and their lineal consanguinities’ physical and mental health are not related to suicidal ideation. These findings imply the different associations between family environment, genetic factors, and suicidal ideation. Family members’ health should be considered as a factor to prevent and control suicidal behaviors, including suicidal ideation.

## 1. Introduction

According to the World Health Organization (WHO)’s estimation, more than 700,000 people die by suicide each year, which means that approximately one person dies by suicide every 40 s in the world [1]. The latest report revealed that the age-standardized suicide rate was 6.7 per 100,000 people in 2019 globally [2]. Suicide not only brings a mental blow to the family but also causes a certain burden of disease and a negative impact on society [3,4,5]. In rural areas, the suicide rate was found to be higher than in urban neighborhoods in many countries around the world [6,7,8]. In China, although the recent suicide rate showed a downward trend [9], suicide is still one of the main causes of death for Chinese rural residents [10]. A study showed that rural areas, where employment and education opportunities were relatively few, accounted for 79 percent of suicides in China [11]. Suicidal ideation (SI), as one kind of suicidal behavior, is the first step of suicidal behavior and a strong predictor of subsequent suicidal behaviors [12,13]. A previous study reported that approximately 13.2% of people who experienced suicidal ideation in the past year attempted suicide [14]. In conclusion, suicidal ideation should be paid close attention to prevent suicide in rural residents.

Many studies have been conducted in recent decades to explore the factors associated with suicidal ideation among different populations [15,16,17,18] as well as the effects of environmental factors on suicide, such as the community and family environment [19,20,21,22,23]. Regarding environmental factors, relatives are closely connected to people’s lives, and their behaviors, experiences, and emotions could affect individuals’ mental health, which had been confirmed in previous studies [24,25,26,27]. While investigating the family consequences of suicide, studies have shown that relatives are affected by suicide and survivors may develop PTSD symptoms and worse mental health [28,29]. In addition to psychological trauma, those survivors also experienced difficulties in social interactions [30]. It was then necessary to provide support from their community, peers, family, and healthcare providers at this time so that they could return to normal life as soon as possible [31,32,33].

In addition, many studies further explored the role of relatives’ characteristics on suicidal ideation. For example, family conflicts, parenting styles, and separated parents were shown to be elements that result in unhealthy emotions and even suicidal behaviors [34,35,36,37]. However, family belongingness and a high educational level of relatives were found to be protective factors against suicidal ideation in different population groups [38,39]. Additionally, support from family was negatively related to suicidal ideation, which is attributed to reducing negative emotions [40,41]. In exploring the influence of relatives, several studies of twins committing suicide and familial cluster suicides confirmed the role of shared environment, showing that among relatives with the same heritability, the more environments they shared and the greater possibility of mutual influence, the higher the risk of suicide [42,43]. Therefore, when considering the effects of relatives on one’s suicidal ideation, the differences among these relatives should be considered. With the shared environment factor confirmed to be associated with suicidal behavior, the associations between living together and suicidal behavior can be assumed. In other words, the connection with suicidal ideation is different between a family member (FM) who lives with the individual and a lineal consanguinity (LC) who does not live with the individual but shares a blood relationship.

Regarding FMs, various studies have found that family characteristics are associated with individuals’ suicidal ideation. Previous studies proved that poor family functioning, family problems, and violence by family members could increase the incidence of suicidal ideation [44,45,46,47]. In addition to these behaviors, several studies indicate that a FM’s health is related to individual suicidal ideation. Concerning physical health, previous literature revealed that severe illness or death of fathers and spouses’ cancer were associated with their FM’s mental status [48]. In China, a study revealed that kids with diagnosed cancer would cause psychological injury to parents, such as desperation and suicidal ideation [49]. In addition to the influence of physical illness, family members suffering from mental illness, such as spouses, parents, and others, may affect the mental status and suicidal ideation of adults [50,51,52]. Alcohol use disorders, as one of the most common mental disorders, result in spousal depression and even suicidal ideation [53]. In addition, another investigation exploring the reasons behind suicidal ideation concluded that relatives’ sickness caused family instability and negative experiences, which could influence different personal life stages and eventually lead to the incidence of negative emotions [4,54,55]. Therefore, the health of FMs has been identified as an influencing factor in suicidal ideation. From another point of view, healthy family members may reduce the probability of suicidal ideation; for example, a suicide management guideline mentioned that the involvement of family members is a protective factor against suicide [56].

For LCs, however, little research has been committed to exploring their effects on suicidal ideation. Only in studies of suicide was the influence of LC’s behavior noticed. For example, a slew of studies showed that suicidal behavior had a tendency for family aggregation [57,58], which means that an attempted or completed suicide in the father’s generation may affect the offspring. In family genetic studies of suicidal behavior, the finding was that impulsive aggregation of a LC may contribute to the familial transmission of suicide [59]. Additionally, another study predicted that the activities or behavior of relatives, such as illegal drug use and aggressive behavior, resulted in a higher risk of suicidal individuals [60]. Hence, the role of LCs in the prevention of suicide has been verified, and it is necessary to further explore its effect on suicidal ideation.

Although the effect of the behavior of a LC on suicide has been identified, few studies have aimed to find the association between a LC’s health and an individual’s suicidal ideation. Furthermore, most studies aimed to observe the influence of relatives’ characteristics on an underage group, rather than adults, but this study focused on the adult population and tried to seek out a new way to prevent adult suicide. In the end, there were few studies examining the differences in the effect of FMs and LCs on suicidal ideation from the perspective of environmental impact. Therefore, the aim of the present study was to investigate the risk factors of suicidal ideation and distinguish the influences of FM’s health and LC’s health with the hope of providing evidence for suicide prevention among rural adults.

## 2. Materials and Methods

### 2.1. Participants and Survey Procedure

The data in this study were collected in November 2019. Respondents of this survey are adults over 18 years of age from the Shandong rural areas in China. Data was collected from Taierzhuang of Shandong province. Shandong, a major province of economy and agriculture, is located in eastern China. Seventeen cities are included in Shandong, and Taierzhuang District, seated in the south of Shandong province, belongs to Zaozhuang City. According to data from the seventh census in China, the resident population of the whole Taierzhuang District is 305,102, and inhabitants residing in rural regions account for 53.72% of the whole population [61]. Five towns in Taierzhuang District were all included in this investigation and a village was randomly chosen from each town. All residents over 18 years old in the village participated in this survey as long as they signed the informed consent. In total, 879 residents were included in this study, and the response rate was 94.9% (879/926).

A face-to-face interview was applied in process of collecting data. Interviewers were postgraduate students who had received professional training and were required to understand the whole research. Before the investigation, respondents and their families agreed to provide relevant information and signed a written informed consent form. As for collecting data, interviewers filled in questionnaires following the answers from the respondents. After the investigation, more than two students inspected the contents and modified the wrong or missing items.

### 2.2. Measures

#### 2.2.1. Suicidal Ideation

The dependent variable of this survey is suicidal ideation. It is evaluated by the question “Have you ever seriously thought over committing suicide?” There existed two answers to this question, “yes” or “no”. Respondents were required to answer this question honestly based on if this thought had ever occurred in their whole life. This question had been used to evaluate suicidal ideation in many previous studies, such as in the exploration of suicidal ideation in college students and adults [20,35].

#### 2.2.2. FM’s and LC’s Health 

According to Chinese traditional habits, a FM refers to relatives living together with individuals currently, and a LC includes relatives who do not live together as adults but have blood relationships, such as uncles, brothers, and sisters. Investigators were asked to explain the differences between two types of relatives in the process of inquiring since the difference between living together and being related needed to be focused on and differentiated.

The health condition of relatives was estimated separately in this study. For FMs, the answer to the question “If your FM suffer from severe chronic diseases?” was used to represent physical health. As for mental health, alcohol use disorders were considered as one mental disorder based on a report from WHO [62]. Therefore, mental health was estimated by two questions. The first question was “If your FM suffer from severe mental illness?” and the other one was “If your FM indulge in excessive drinking?”. There were two answers to all questions, “yes” or “no”. For LCs, the inquiries were similar to the questions for FMs. Regarding physical health, this study used the question “If your LC suffer from severe chronic diseases?” for estimation. Mental health was addressed using the two questions, “If your LC suffer from severe mental illness?” and “If your LC indulge in excessive drinking?”.

#### 2.2.3. Socio-Demographic Characteristics

The questionnaire also involved basic socio-demographic information, such as gender, age, occupation, marriage status, education level, and family composition. Gender was divided into two categories: male and female. Age was obtained by subtracting the year of birth from the current year and classified as 18–40 and >40 years old. Occupation types included farmers, students, personnel of public institutions, businessmen, and so on, but the occupation was reclassified into two categories, “freelancer” and “others”, for the convenience of data analysis. Marriage status was represented by married and other conditions “unmarried, divorced and widowed”. Considering the low educational level in rural areas, junior high school education was selected as the dividing line, and results were classified into primary school education and below and junior high school education level and above. The familial composition was evaluated by one question “Were you an only child?” 

#### 2.2.4. Household Indebtedness and Difficulty Falling Asleep

In addition to the above variables, household indebtedness and sleeping difficulty were also risk factors. The household indebtedness condition was estimated by the question “Was your family in debt?”, with two choices of answers, “yes” or “no”. Cannot fall asleep within 30 min was defined as difficulty falling asleep according to the PSQI, and there were four levels of the answer, “not”, “once a week”, “twice a week”, and “three times a week and above”. Similarly, we grouped the answers into two categories “yes” or “no” in the analysis of data.

### 2.3. Data Management and Analysis

IBM SPSS Statistics 26.0 was the main software for analyzing data. The Chi-square test was used to describe social-demographic characteristics and discover the single risk factors of suicidal ideation. Logistic regression analysis, differing from single-factor analysis, was applied to examine the effects of multiple independent variables on suicidal ideation. We coded all answers as “1” and “0” and considered the results to be statistically significant when the *p*-value was less than 0.05.

## 3. Results

### 3.1. Demographic Characteristics and Suicidal Ideation of the Sample

Table 1 presents the demographic characteristics of the sample, and a single-factor analysis for suicidal ideation was included in this table. A total of 879 sample cases were conducted in this study, of which, 322 were males and 557 were females. Results showed that 60 (6.83%) cases of rural residents had experienced suicidal ideation. 

**Figure 1:**
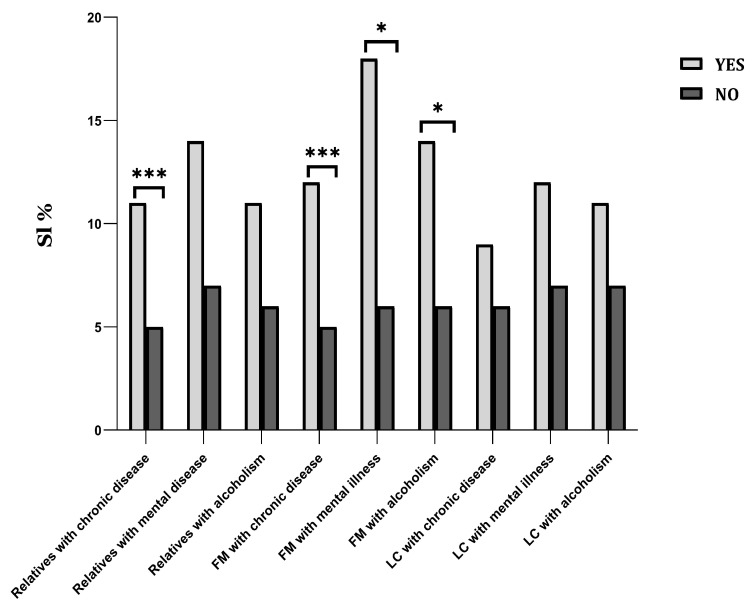
Visual comparisons for suicidal ideation under different relatives’ characteristics. Note: FM = Family member, LC = lineal consanguinity, and SI = suicidal ideation; * *p* < 0.05, *** *p* < 0.001.

In Figure 1, for all the relatives, the results of the single-factor analysis indicated that there existed no significant differences in the incidence of suicidal ideation in relatives whether with mental illness and with alcoholism or not. According to the results, relatives with chronic disease (4.87% vs. 10.92%, *p* < 0.001) were associated with suicidal ideation. After classification, however, only having a FM with a chronic disease (5.16% vs. 11.82%, *p* < 0.001), mental illness (6.46% vs. 17.86%, *p* < 0.05), or alcoholism (6.39% vs. 14.00%, *p* < 0.05) were considered as risk factors.

Regarding other factors in Table 1, the results of the single-factor analysis indicated that there existed no significant differences between different sexes, occupations, and marital statuses. The population over 40 years old, compared to the 18–40 population (8.17% vs. 1.18%, *p* < 0.001), had a higher prevalence of suicidal ideation. While comparing people with a junior high school degree and above (8.66% vs. 3.51%, *p* < 0.01), those with fewer years of education had a higher prevalence of suicidal ideation. Comparisons were also conducted between only-child families and multiple-child families; the result showed that people from only-child families (6.38% vs. 18.18%, *p* < 0.01) had a higher incidence of suicidal ideation. Regarding other conditions, risk factors included household indebtedness (4.66% vs. 12.06%, *p* < 0.001) and falling asleep with difficulty (3.37% vs. 12.17%, *p* < 0.001).

### 3.2. Logistic Regression Analysis of Suicidal Ideation

For further analysis of factors related to suicidal ideation in rural residents, suicidal ideation as the dependent variable, and demographic characteristics, sleep status, and relatives’ health as the independent variables, were included in Model 1 of the logistic regression analysis. All of the results are shown in Table 2. Results showed that an age > 40 (OR = 5.525, *p* = 0.026), from an only-child family (OR = 4.511, *p* = 0.004), household indebtedness (OR = 2.899, *p* < 0.001), difficulty falling asleep (OR = 3.057, *p* < 0.001), and relatives with chronic disease (OR = 2.051, *p* = 0.012) were associated with suicidal ideation. In Model 2, we classified the relatives further and included the variables on FM’s health and LC’s health. The results showed that an age >40 (OR = 6.528, *p* = 0.018), from an only child family (OR = 4.335, *p* = 0.006), household indebtedness (OR = 2.992, *p* < 0.001), difficulty falling asleep (OR = 3.165, *p* < 0.001), and a FM with chronic illness (OR = 2.303, *p* = 0.008) or mental illness (OR = 5.877, *p* = 0.028) were risk factors of suicidal ideation.

## 4. Discussion

In this study, the main objective was to identify the influencing factors of suicidal ideation and differentiate the effect of FMs’ and LCs’ health on suicidal ideation among rural adults. Results showed that the prevalence of suicidal ideation in rural residents was 6.83% in this survey, which was close to the estimation of other studies, such as research in rural areas of Shandong Province and Zhejiang Province [63,64,65]. The relevant risk factors resulting in suicidal ideation included an age >40, an only child family, household indebtedness, and difficulty falling asleep. Concerning the effect of relatives’ health, the association between relatives with chronic disease and suicidal ideation was confirmed. The influences of relatives were then discussed from two aspects: FMs’ health and LCs’ health. To be specific, FMs’ health was more related to suicidal ideation compared to LCs’ health, and adults whose FMs had a chronic disease or mental illness were at a higher risk for suicidal ideation. For rural residents, family characteristics could affect one’s mental status [66,67]; the role of a FM’s health on suicidal ideation was proved in a study in rural Haiti, which was consistent with this result [68]. This could be because rural residents live in distant areas that lack transportation and sufficient income [69], and a FM’s illness may lead to increased pressure on individuals on account of less social service available.

Collectively, this study observed that relatives with chronic disease were associated with individuals’ suicidal ideation firstly, which was consistent with results concluded by previous research. Regarding this, relatives’ physical health was determined to be a risk factor affecting suicidal ideation in past studies [48,49]. The reasons for this relevance cause concern and explorations were presented in numerous studies. For example, a previous survey explained that a person developed fear or anxiety about losing a loved one after a relative was diagnosed with a pathogenic chronic disease [70]. Another study suggested that negative emotions from diagnosed relatives would cause an individual passive psychological experience [71]. Further analysis explaining this reason indicated that these passive psychological experiences were predictors of suicidal ideation, such as depression, anxiety, and hopelessness, in past findings [72,73,74]. Hence, the effect of the physical health of relatives on suicidal ideation was well established, and further exploration of relatives’ health needs to be carried out on this basis.

In the comparison of FMs and LCs, we found that FMs who suffer from chronic disease and mental illness had a strong connection with suicidal ideation, but a LC’s health was not associated with it. The greatest distinction between FMs and LCs lay in whether they live with the individual, that is, FMs share the living environment and LCs do not. Further exploration demonstrated that a safe family, public, and hospital environment were necessary for the prevention of suicide in adults. Relatedly, the environment was found to be a factor associated with the suicide rate of the elderly [75,76]. Studies found that adults would feel anxious and stressed while living together with diseased relatives [77], and even hopelessness or anxiety could be produced in the process of care and treatment [78]. Meanwhile, a previous result also showed that a FM’s chronic disease had a great connection with the negative emotions of adults, and unfavorable economic situations increased opportunities for one’s emotional distress [79]. Therefore, a FM’s chronic disease and mental illness could lead to suicidal ideation by means of the environment and negative emotions. However, there was no significant association between suicidal ideation and relatives with alcoholism in this study, whether in FMs or LCs. Previous studies supported that parental alcoholism had no direct impact on students’ suicidal ideation [80,81]. However, other hypotheses verified that spousal alcohol abuse may result in women’s suicidal behaviors [82,83]. As a result, further investigation into the effect of a FM’s or LC’s alcoholism needs to be launched and discussed.

Regarding other factors, the present study also found that there was a greater possibility of suicidal ideation in people over 40 years old. Previous studies also indicated that the prevalence of suicidal ideation in the middle-aged and elderly group was associated with loneliness, household financial problems, and poor perceived health [84,85]. This study also revealed that the prevalence of suicidal ideation was higher in people from only-child families, which was consistent with a previous result [86]. Experts explained that elderly people from rural areas, with a lack of pension provision and children, generally had a sense of responsibility to their parents, which resulted in an increase in the stress of life as an only child [87]. Therefore, more care should be given to rural adults for preventing suicide. This finding also demonstrated that household indebtedness was a risk factor influencing adults’ suicidal ideation. Similarly, a previous study mentioned that people with increased economic pressure caused by over-indebtedness were in danger of mental health problems, and the connection between the two varied depending on the debt amount and debt sources [88,89]. Difficulty falling asleep was identified as significant in this study of factors affecting suicidal ideation, which is consistent with the conclusions from previous studies [90,91]. A study of Australian adults showed that difficulty falling asleep, as one specific aspect of sleep disorder, strongly influenced the prevalence of suicidal ideation and played an independent role [92].

Collectively, a FM’s physical and mental health status, as one kind of factor of the living environment, could affect an individual’s mood and behaviors. Specific reasons may be the stress as a caregiver and negative emotions caused by the disease while living together. Furthermore, people over 40 years old, people from only-child families, people with household indebtedness, and people with difficulty falling asleep were considered to be in high-risk groups who were more likely to have suicidal ideation.

In this study, certain limitations should be considered. Firstly, the study only opened an investigation and collected data from rural areas of Taierzhuang District, and we should be cautious when interpreting the findings into other populations and regions. Secondly, the data of this study came from the self-reporting of respondents, which may have recall bias. Next, although on a large scale, differences between the effect of FMs and LCs on suicidal ideation were explored, but exact reasons for these differences were not evidenced in this study. Finally, the present study did not discover the effects of FMs’ and LCs’ alcoholism on suicidal ideation in adults, so further validation is required in future studies.

## 5. Conclusions

In conclusion, the prevalence of suicidal ideation in Shandong rural adults was 6.83%. Before categorization, only relatives with chronic disease were a risk factor for suicidal ideation. Comparing the influences of relatives’ health, results indicated that FMs who suffered from chronic disease and mental illness made an impact on suicidal ideation, and an evident correlation was not found in LCs. This difference reminded us that the psychological condition of adults with a FM suffering from health disorders should be monitored closely. In addition to studying the impact of relatives’ health on suicidal ideation, there needs to be more attention paid to people over 40 years old, people from only-child families, people with household indebtedness, and people with difficulty initiating sleep.

Focusing on adults with a FM suffering from physical and mental illness and publicizing the influence of the living environment on suicidal ideation are crucial. Hence, more measures need to be taken for reducing suicidal ideation by the melioration of FMs’ physical and mental health. More action needs to be taken in the community after identifying the impact of family members’ health on an individual’s suicidal ideation. The government should encourage the community to pay more attention to the mental health of a patient’s family members for the purpose of suicide prevention. Further, doctors need to focus on the role of relatives in the treatment of suicide, in order to reduce the suicide rate of rural residents, make suicide prevention and intervention plans based on the home environment, provide more social and psychological services, and improve family happiness for rural adults.

## Figures and Tables

**Table 1:** Characteristics and univariate analysis of suicidal ideation among the samples.

Variables	Total	Suicidal Ideation		χ^2^
		Yes	No	
N	879 (100)	60 (6.83)	819 (93.17)	
Age				10.474 ***
18–40	169 (19.23)	2 (1.18)	167 (98.82)	
>40	710 (80.77)	58 (8.17)	652 (91.83)	
Gender				0.684
Male	322 (36.63)	19 (5.90)	303 (94.10)	
Female	557 (63.37)	41 (7.36)	516 (92.64)	
Occupation				0.958
Freelancer	737 (83.85)	53 (7.19)	684 (92.81)	
Other	142 (16.15)	7 (4.93)	135 (95.07)	
Marital status				2.417
Married	749 (85.21)	47 (6.28)	702 (93.72)	
Other	130 (14.79)	13 (10.00)	117 (90.00)	
Education Level				8.382 **
Primary school education and below	566 (64.39)	49 (8.66)	517 (91.34)	
Junior high school education and above	313 (35.61)	11 (3.51)	302 (96.49)	
Family composition				6.952 **
Multiple-child	846 (96.25)	54 (6.38)	792 (93.62)	
Only child	33 (3.75)	6 (18.18)	27 (81.82)	
Household indebtedness				15.658 ***
Yes	257 (29.24)	31 (12.06)	226 (87.94)	
No	622 (70.76)	29 (4.66)	593 (95.34)	
Difficulty falling asleep				25.538 ***
Yes	345 (39.25)	42 (12.17)	303 (87.83)	
No	534 (60.75)	18 (3.37)	516 (96.63)	
Relatives with chronic disease				11.033 ***
Yes	284 (32.31)	31 (10.92)	253 (89.08)	
No	595 (67.69)	29 (4.87)	566 (95.13)	
Relatives with mental disease				
Yes	37 (4.21)	5 (13.51)	32 (86.49)	2.716
No	842 (95.79)	55 (6.53)	787 (93.47)	
Relatives with alcoholism				2.264
Yes	72 (8.19)	8 (11.11)	64 (88.89)	
No	807 (91.81)	52 (6.44)	755 (93.56)	
FM with chronic disease				11.499 ***
Yes	220 (25.03)	26 (11.82)	194 (88.18)	
No	659(74.97)	34 (5.16)	625 (94.84)	
FM with mental disease				5.534 *
Yes	28 (3.19)	5 (17.86)	23 (82.14)	
No	851 (96.81)	55 (6.46)	796 (93.54)	
FM with alcoholism				4.290 *
Yes	50 (5.69)	7 (14.00)	43 (86.00)	
No	829 (94.31)	53 (6.39)	776 (93.61)	
LC with chronic disease				2.076
Yes	159 (18.09)	15 (9.43)	144 (90.57)	
No	720 (81.91)	45 (6.25)	675 (93.75)	
LC with mental disease				1.083
Yes	25 (2.84)	3 (12.00)	22 (88.00)	
No	854 (97.16)	57 (6.67)	797 (93.33)	
LC with alcoholism				1.500
Yes	44 (5.01)	5 (11.36)	39 (88.64)	
No	835 (94.99)	55 (6.59)	780 (93.41)	

Note: FM = Family member, LC = Lineal consanguinity; * *p* < 0.05, ** *p* < 0.01, *** *p* < 0.001.

**Table 2:** Logistic regression analysis of risk factors associated with suicidal ideation among rural adults {OR (95%CI)}.

Independent Variables	Model 1	Model 2
Age > 40	5.525 (1.225~24.925) *	6.528 (1.384~30.795) *
Female	1.111 (0.595~2.075)	1.145 (0.603~2.176)
Freelancer	0.863 (0.350~2.126)	0.779 (0.313~1.937)
Married	0.755 (0.378~1.508)	0.708 (0.351~1.429)
Junior high school education and above	0.715 (0.323~1.582)	0.791 (0.351~1.781)
Only child	4.511 (1.602~12.700) **	4.335 (1.529~12.295) **
Household indebtedness	2.899 (1.661~5.063) ***	2.992 (1.704~5.256) ***
Difficulty falling asleep	3.057 (1.659~5.634) ***	3.165 (1.699~5.897) ***
Relatives with chronic disease	2.051 (1.170~3.597) *	——
Relatives with mental disease	1.957 (0.669~5.727)	——
Relatives with alcoholism	1.432 (0.612~3.350)	——
FM with chronic disease	——	2.303 (1.244~4.267) **
FM with mental illness	——	5.877 (1.214~28.446) *
FM with alcoholism	——	2.405 (0.781~7.406)
LC with chronic disease	——	0.989 (0.476~2.057)
LC with mental illness	——	0.503 (0.081~3.128)
LC with alcoholism	——	0.858 (0.243~3.035)

Note: FM = Family member and LC = lineal consanguinity; * *p* < 0.05, ** *p* < 0.01, and *** *p* < 0.001. —— means the variable is not considered in Model.

## Data Availability

The data presented in this study are available on request from the corresponding author. The data are not publicly available due to the privacy of survey respondents.
